# Cardiometabolic risk assessment by anthropometric and biochemical indices in mexican population

**DOI:** 10.3389/fendo.2025.1588469

**Published:** 2025-07-14

**Authors:** Luz Quirino-Vela, Miguel Mayoral-Chavez, Yobana Pérez-Cervera, Osiris Ildefonso-García, Elizabeth Cruz-Altamirano, Monserrat Ruiz-García, Juan Alpuche

**Affiliations:** ^1^ Centro de Investigación Facultad de Medicina UNAM-UABJO, Facultad de Medicina y Cirugía, Universidad Autónoma Benito Juárez de Oaxaca, Oaxaca, Mexico; ^2^ Centro de Estudios en Ciencias de la Salud y la Enfermedad, Facultad de Odontología, Universidad Autónoma Benito Juárez de Oaxaca, Avenida Universidad S/N, Oaxaca de Juárez, Oaxaca, Mexico

**Keywords:** cardiometabolic risk, anthropometric indices, biochemical indices, obesity, mexican adults

## Abstract

**Background:**

Cardiometabolic risk (CMR) factors, including obesity, hypertension, hyperglycemia, and dyslipidemia, are major contributors to global morbidity and mortality. Although gold-standard diagnostic methods for obesity and insulin resistance exist, they are costly and inaccessible in resource-limited settings. Conventional anthropometric measures underestimate parameters that enhance risk prediction and fail explaining the complex relationship between adipose tissue distribution and metabolic dysfunction. This study evaluated and compared the diagnostic accuracy of 15 conventional and non-conventional anthropometric and biochemical indices for identifying CMR factors in Mexican adults. We hypothesized that non-conventional indices would demonstrate superior diagnostic performance compared with traditional measures.

**Methods:**

We analyzed data from 1,876 participants aged 20–80 years from the 2022 National Health and Nutrition Survey (ENSANUT). Anthropometric indices, including body mass index (BMI), waist-to-height ratio (WHtR), body roundness index (BRI), deep abdominal adipose tissue index (DAAT), and weight-adjusted waist index (WWI), were calculated alongside biochemical indices such as HOMA-IR, triglyceride-glucose index (TG), and combined indices like TG*BMI and TG*WC. Receiver operating characteristic (ROC) curve analysis evaluates diagnostic performance, with sex-stratified analyses conducted to determine optimal cut-off values.

**Results:**

Non-conventional indices demonstrated superior diagnostic performance across all outcomes. For obesity detection, TG*BMI achieved the highest area under the curve (AUC=0.972), followed by WHtR and BRI (AUC=0.934). For CMR assessment, BRI showed perfect discrimination (AUC=1.000), whereas TG*WC (AUC=0.976) and LAP (AUC=0.963) demonstrated exceptional performance. Sex-based analyses revealed that optimal cut-off values varied, with most indices maintaining a consistent discriminatory capacity across sexes.

**Conclusions:**

Non-conventional anthropometric indices, particularly those incorporating metabolic and anthropometric parameters, outperform traditional BMI-based assessments for CMR stratification in Mexican adults. These accessible, cost-effective tools offer superior diagnostic accuracy and enhance early identification of high-risk individuals in resource-limited healthcare settings. Future studies are required to validate these findings and establish population-specific reference values.

## Highlights

- The anthropometric indices WHtR, BRI, and DAAT showed the best correlation with cardiometabolic risk factors.
- Biochemical indices TG, TG/HDL, and AIP had higher correlations than HOMA-IR and QUICKI.
- TG*BMI, WHtR, BRI, TG*WC, DAAT, and LAP had higher AUC values for detecting obesity.
- BRI, TG*WC, LAP, BMI, TG*BMI, DAAT, and WWI had higher AUC values for assessing cardiometabolic risk, and TG*BMI and TG*WC had higher AUC values for evaluating insulin resistance.
- Anthropometric indices performed better than biochemical indices in assessing cardiometabolic risk factors.

## 1 Background

Cardiometabolic risk (CMR) encompasses modifiable factors that synergistically increase vascular events and metabolic dysfunction (1–3), including abdominal obesity, hypertension, hyperglycemia, dyslipidemia, and lifestyle factors that collectively damage cardiac and vascular structures, elevating cardiovascular disease (CVD) risk, leading global mortality cause (4, 5). Obesity is a major cardiometabolic risk factor characterized by pathological adipose accumulation that increases white adipose tissue (WAT) and is associated with various metabolic disorders (6–9). Visceral adiposity secretes the pro-inflammatory cytokines interleukin-6 (IL-6) and tumor necrosis factor-α (TNF-α), reducing endothelial nitric oxide and upregulating adhesion molecules that initiate atherosclerosis (10). Moreover, increased free fatty acids (FFAs) release from adipose tissue disrupts insulin signaling, promotes hepatic glucose production, and activates cholesteryl ester transfer protein, thereby disrupting vascular homeostasis and, exacerbating atherogenic dyslipidemia and inflammation (11).

Visceral adipocyte dysfunction promotes insulin resistance, dyslipidemia, hypertension, and hyperglycemia through impaired lipid metabolism and vascular regulation (12). In patients with type 2 diabetes (T2D), obesity, and atherogenic dyslipidemia promote vascular obstruction, elevating coronary heart disease risk and mortality from cardiovascular, renal, and infectious diseases (11, 13, 14). Additionally, triglycerides in HDL particles are the primary factors for hypertension, whereas atherogenic lipoproteins increase the pulse pressure (15). Additionally, obesity-mediated WAT dysfunction triggers dysregulated oxylipin pathways, particularly reduced cytochrome P450-soluble epoxide hydrolase activity during metabolic syndrome, compromising tissue homeostasis and inflammation reduction (16). Brown adipose tissue (BAT) activation offers promising interventions by enhancing thermogenesis, improving metabolic parameters, and strengthening intestinal barrier integrity while reducing inflammation (17). The convergence of abdominal obesity, hypertension, dyslipidemia, insulin resistance, and inflammation create a self-reinforcing cycle of metabolic-vascular dysfunction, emphasizing the need for integrated cardiometabolic assessment guiding risk stratification and clinical intervention.

Gut microbiota composition may predict responses to dietary and pharmacological interventions for personalized cardiometabolic therapies (18). Multi-omics research demonstrated microbiota–metabolite signatures significantly impact metabolic development, highlighting microbiome contributions to CMR (19).

The gold standards for diagnosing metabolic diseases are computed tomography (CT) and magnetic resonance imaging (MRI), which accurately quantify adipose tissue; however, their complexity and high cost limit their routine use. Although MRI is radiation-free, it is time-consuming and expensive (20, 21). The hyperinsulinemic-euglycemic clamp (HEC), reference method for insulin resistance assessment, requires specialized staff, equipment, and multiple arterial samples, restricting large-scale application (22). Echocardiography aids cardiac evaluation but is operator-dependent, and inexperience can affect the accuracy of diagnosis (23, 24).

Anthropometric indices are accessible, noninvasive, and cost-effective tools for cardiometabolic risk assessment across diverse populations (25). While traditional measures have been widely implemented, emerging evidence supports the superior utility of non-conventional indices in capturing the complex relationship between adiposity distribution and metabolic dysfunction (10, 26–31).

Traditional anthropometric indices -including waist circumference (WC), waist-to-height ratio (WHtR), and waist-to-hip ratio (WHR)- have been extensively utilized to evaluate general or central obesity, however, these conventional measures often overlook potentially valuable parameters that could enhance risk prediction and fail to differentiate between adipose tissue types, despite the critical influence of fat distribution on obesity-related outcomes (32, 33). Conventional anthropometric indices offer valuable approaches to cardiometabolic risk assessment, with body mass index (BMI) and other traditional metrics remaining associated with long-term cerebrovascular events and providing estimates of insulin resistance (14, 34, 35). However, these indices frequently fail to capture metabolic heterogeneity among individuals with similar BMI values.

The distinction between adipose tissue compartments is particularly relevant, as visceral adipose tissue promotes a proinflammatory state and significantly increases the risk of atherosclerosis, cardiometabolic diseases, T2D, and cardiovascular events (36–38). Consequently, accurate evaluation of visceral adiposity has become imperative for comprehensive cardiometabolic risk assessment (32).

Several non-traditional indices effectively assess visceral adiposity, each selected for this study based on their unique physiological and methodological advantages. The A Body Shape Index (ABSI) quantifies abdominal obesity and sarcopenic obesity (39, 40), while evaluating visceral fat linked to cardiovascular morbidity, predicting outcomes not captured by conventional metrics, including all-cause mortality, metabolic syndrome, diabetes, and hypertension (41–44). The Body Roundness Index (BRI) estimates total adiposity using an elliptical modeling approach that better approximates variations in human body shape than simple circumference measurements or BMI calculations, effectively predicting metabolic syndrome (MetS) and CMR (26). The index correlates strongly with visceral adiposity markers and has demonstrated superior association with arterial stiffness, metabolic syndrome, and hypertension compared to traditional measures (45–47). The deep abdominal adipose tissue index (DAAT) can directly estimate visceral fat accumulation and the predictability of cardiovascular events by incorporating mathematical modeling that incorporates weight, waist circumference, and BMI into sex-specific equations, specifically targeting the metabolically active visceral adipose tissue compartment that conventional indices cannot distinguish (27, 32). This index has demonstrated a predictive capacity for cardiovascular events by capturing the inflammatory and metabolic dysfunctions associated with visceral fat accumulation (48). The Weight-adjusted waist circumference index (WWI) is a strong predictor of cardiovascular morbidity, mortality, and hypertension, consistently outperforming traditional measures such as BMI, WC, ABSI, and WHR in diverse populations (28, 49–51). WWI evaluates both central adiposity and overall body composition by adjusting waist circumference for weigh, capturing a phenotype associated with increased cardiovascular mortality, particularly valuable in populations with sarcopenic obesity where conventional indices may underestimate cardiometabolic risk (28, 50, 51).

Complementing the properties of non-conventional indices, combined anthropometric-laboratory indices such as the Visceral Adiposity Index (VAI), which combines WC, BMI with TG and HDL, surrogates of insulin resistance, and cardiometabolic risk (13). VAI combines anthropometric measurements (waist circumference, BMI) with lipid parameters (triglycerides, HDL cholesterol) in sex-specific formulas designed to reflect visceral adipose function, correlating with CMR, hypertension, insulin resistance, and albuminuria (29, 39, 52, 53). Lipid Accumulation Product (LAP) combines waist circumference (WC) and triglyceride levels, which reflects pathophysiological processes underlying cardiometabolic risk, particularly insulin resistance and pre-diabetes (30, 31), demonstrating greater efficacy in predicting prediabetes and incident T2D (45, 54, 55), surpassing BMI in CMR prediction (30, 31). The integration of anthropometric and lipid markers achieves a more precise CMR assessment than anthropometry alone. Indeed, TG alone serves as a strong predictor of prediabetes and in combination with BRI, VAI, or ABSI, significantly enhances the diagnostic accuracy and prediction of stroke incidence (56, 57). TG*BMI and TG*WC have superior performance in detecting insulin resistance and cardiometabolic risk. The combination of TG levels and anthropometric measures simultaneously captures both metabolic dysfunction and adiposity distribution. This dual approach provides a more comprehensive cardiometabolic risk assessment than either component alone (14, 35). Moreover, the triglyceride-to-cholesterol ratio, triglyceride-to-glucose ratio to high-density lipoprotein cholesterol (TG/HDL-c) associates to arterial stiffness progression (58).

Biochemical blood parameters play a crucial role in assessing cardiometabolic risk, providing valuable insights into metabolic health and cardiovascular disease risk. These parameters typically include lipid profiles (total cholesterol, LDL cholesterol, HDL cholesterol, and triglycerides), glucose levels, and glycated hemoglobin (HbA1c) levels. The insulin resistance (IR) related to glucose and insulin levels, is a key factor for metabolic syndrome, obesity, and cardiovascular diseases (59, 60). The homeostasis model of insulin resistance (HOMA-IR) evaluates IR and the pancreatic beta cell function, and the quantitative insulin sensitivity check index (QUICKI) which evaluates the ability of cells to respond to the effect of insulin, offer accessible and effective ways to determine IR (30, 31, 61).

While prior studies have validated conventional indices (BMI, WHtR) and developed Mexican-specific tools (MAIs/BAIs) for assessing cardiometabolic risk (62–65), critical gaps persist regarding the comparative efficacy of non-conventional anthropometric indices—both independently and in combination with biochemical parameters—within Mexico’s unique demographic context. Rodriguez-Carrillo et al. (2021) advanced this field by establishing sex-specific MAIs and BAIs to identify visceral adiposity and metabolic syndrome (66–69), yet their work did not systematically compare these tools against emerging non-conventional indices (e.g., BRI, WWI, DAAT) or evaluate their integrated use with biochemical markers. This omission leaves unresolved whether novel indices improve risk detection over conventional measures in Mexican adults, particularly given population-specific adiposity patterns and metabolic profiles. To address this, we calculated 15 indices and hypothesized that non-conventional anthropometric indices would demonstrate stronger correlations with biochemical parameters and superior diagnostic performance for cardiometabolic risk factors than traditional measures. This comparative analysis fills a critical gap in optimizing risk stratification strategies for Mexico’s high-risk population.

## 2 Methods

### 2.1 Data collection

We obtained data on participants from the National Health and Nutrition Survey, 2022 (ENSANUT) database (Publicly available at: https://ensanut.insp.mx/encuestas/ensanutcontinua2022/descargas.php.) ENSANUT is a national multistage probability sampling survey that was conducted between July 28, 2022, and December 10, 2022, encompassing 14,240 households, to obtain 10,450 households with complete information from eight regions of Mexico (Pacific North, Border, Pacific Central, Pacific North, Central, Mexico City State of Mexico, Pacific South, and Peninsula) (70). The ENSANUT is a structured questionnaire that collects basic information (age, sex, unit), anthropometric measurements of weight (kg), height (cm), and waist circumference (cm), as well as blood pressure (mmHg). Anthropometric measurements were performed in duplicate. Blood pressure (mmHg) was measured using a standard mercury sphygmomanometer.

Fifteen milliliters of blood were collected from each participant after fasting via venous puncture of the forearm to obtain laboratory glucose, cholesterol, triglycerides, HDL, and LDL levels. National Institutes, Instituto Nacional de Diagnóstico y Referencia Epidemiología Dr Manuel Martínez Báez (InDRE), Ciencias Médicas y Nutrición Salvador Zubirán (INCMNSZ), and Salud Pública (INSP) laboratories analyzed the samples. Trained personnel collected biological samples and performed anthropometric measurements. The INSP Ethics, Research, and Biosafety Commission authorized the questionnaires, interviews, and informed consent forms. The participants had to accept and sign an informed consent form.

### 2.2 Participants

After acquiring the database, we filtered the data to include only men and women aged 20–80 years. The initial sample comprised 6,833 individuals with biochemical test results; however, patients with risk factors that could influence the effect of the anthropometric indices were excluded from the study. These risk factors include the diagnosis of type 2 diabetes (T2D), diagnosis of gestational diabetes (GDM), lower and upper limb amputations, and pregnancy. Furthermore, participants who failed to undergo complete anthropometric measurements or biochemical tests, or those with incomplete general data, were excluded from the final analysis. Only participants whose information was complete and validated in the database were included. This avoids bias derived from missing or inconsistent data. The final analysis included 1,876 participants.

#### 2.2.1 Classification of participants

The participants were classified into two groups according to CMR, defined by central obesity status using WHtR (cut-off of ≥0.50, indicating CMR presence). This index has shown superior performance over other indices in detecting central adiposity and associated metabolic dysregulation (66–69). While CMR conventionally encompasses multiple components (e.g., dyslipidemia, hypertension), our operational definition focused on central obesity because of its established role as a primary driver of cardiometabolic pathophysiology and its feasibility in resource-limited settings. The selected WHtR cut-off has been extensively validated across diverse populations for identifying individuals at elevated risk of insulin resistance, dyslipidemia, and cardiovascular events (71–74), ensuring its applicability to Mexico’s clinical infrastructure.

### 2.3 Anthropometric measurements

Anthropometric indices were calculated using previously established methodologies.

1. Body mass index, $BMI=\frac{weight}{height^{2}}$ (75).
2. Waist-to-height ratio, $WHtR=\frac{WC}{height}$ (76).
3. A body shape index, $ABSI=\frac{WC}{\left(height_{2}^{1}\times BMI_{3}^{2}\right)}$ (77).
4. Body roundness index, $BRI=364.2-365.5\sqrt{1-\frac{{\left(\frac{wc}{2\pi }\right)}^{2}}{{\left(0.5\times height\right)}^{2}}}$ (78).
5. Deep abdominal adiposity tissue index, DAAT (48).

$$\begin{array}{c} Males=-382.9+(1.09\times weight-(kg))+(6.04\times WC-(cm))+\\ \left(-2.29\times IMB(kg/m^{2})\right) \end{array}$$

Females=−278+(−0.86×weight−(kg))+(5.19×WC−(cm))

6. Weight-adjusted-waist index, $WWI=\frac{WC}{\sqrt{weight}}$ (49).

### 2.4 Biochemical measurements

Indices related to biochemical parameters, including HOMA-IR, TG, TG/DHK, QUICKI, and AIP, were calculated using the following methods:

7. Homeostasis Model Assessment insulin resistance index, HOMA-IR (79).

HOMA−IR=Fasting glucose(mmol/L)×fasting inulin(UI/ml)/405

8. Triglycerides to Glucose Index, TG (80).

TG=Ln [fasting triglycerides (mg/dL)×fasting plasma glucose (mg/dL)/2]

9. Triglycerides to high-density lipoprotein cholesterol ratio, TG/HDL-C (81).

TG/HDL=ratio [TG (mmol/dL) divided by HDL−C (mmol/dL)]

10. Quantitative insulin sensitivity check index, QUICKI (82).

QUICKI=(1/[log fasting insulin (μU/mL)+log fasting glucose (mg/dL)])

11. Atherogenic index of plasma, AIP (13).

AIP=Log10[Tg(mmol/L)/HDL (mmol/L)]

### 2.5 Biochemical indices with anthropometric measurements

The VAI, LAP, TyG*BMI, and TyG*WC indices were calculated as follows:

12. Visceral adiposity index, VAI (83).

$$\begin{array}{c} Males=WC\;(cm)/[39.68+1.88\times BMI\;(kg/m^{2})]\\ \times [TG\;(mmol/L)/1.03]\times [1.31/HDL\;(mmol/L)]. \end{array}$$

$$\begin{array}{c} Females=WC\;(cm)/[36.58+1.89\times BMI\;(kg/m^{2})]\\ \times [TG\;(mmol/L)/0.81]\times [1.52/HDL\;(mmol/L)]. \end{array}$$

13. Lipid accumulation product, LAP (84).

Male sex=[WC (cm)−65]×TG (mmol/L).

Female=[WC (cm)−58]×TG (mmol/L).

14. Trigycerides to Body mass index, TyG*BMI (14).

$$\begin{array}{c} Ln\;[fasting\;triglycerides(mg/dL)\\ fasting\;plasma\;glucose(mg/dL)/2]\times BMI \end{array}$$

15. Triglycerides to Waist circumference. TyG*WC (13).

$$\begin{array}{c} Ln\;[fasting\;triglycerides\;(mg/dL)\\ fasting\;plasma\;glucose\;(mg/dL)/2]\times WC \end{array}$$

### 2.6 Statistical analysis

Data analysis was conducted using IBM SPSS Statistics version 23. A p-value (bilateral) <0.05 indicated statistical significance; continuous numerical variables were presented as mean and standard deviation, Student’s t-test for continuous variables, Pearson’s correlation test for continuous numerical variables, and were prepared by plotting 1-specificity on the x-axis and sensitivity on the y-axis to evaluate the prognostic capacity of biochemical, anthropometric, and biochemical-anthropometric indices. The cut-off point value that had the highest Youden index was selected as the optimal cut-off point for each index (Youden index=sensitivity+specificity-1).

## 3 Results

A total of 1,876 participants were included, comprising 40.2% men and 59.8% women, with a mean age of 43.50 (± 15.01) years. Participants were stratified by cardiometabolic risk (CMR) status using WHtR ≥0.50, resulting in 1,342 individuals with CMR (71.5%) and 534 without CMR (28.5%). The BMI was higher in women (29.97 kg/m2), approaching the obesity threshold of 30 kg/m2, whereas in men, it was 28.65 kg/m2, placing them in the overweight category (p<0.001). The LAP, ABSI, DAAT, WWI, TG, TG*WC, TG/HDL, and AIP indices were significantly higher in men than in women, while women had higher BRI and WHtR values (p<0.001) **Table 1**.

**Table 1:** Baseline characteristics stratified by sex and cardiometabolic risk status in mexican adults (N=1,876).

Variable	Total (n=1,876)	Male (n=754)	Female (n=1,122)	p value	With CMR (n=1,697)	Without CMR (n=179)	p value
Age (years)	43.50 ± 15.01	44.36 ± 15.99	42.92 ± 14.30	0.046	44.55 ± 14.71	33.52 ± 14.21	0.000
Height (cm)	158.78 ± 9.58	166.50 ± 7.38	153.60 ± 7.07	0.000	158.27 ± 9.54	163.63 ± 8.59	0.000
Weight (kg)	74.34 ± 16.84	79.68 ± 17.50	70.75 ± 15.38	0.000	76.13 ± 16.27	57.38 ± 12.01	0.000
WC (cm)	96.76 ± 13.70	98.49 ± 13.96	95.61 ± 13.40	0.000	99.12 ± 12.07	74.47 ± 5.94	0.000
WHtR	0.61 ± 0.08	0.59 ± 0.08	0.62 ± 0.09	0.000	0.62 ± 0.07	0.45 ± 0.03	0.000
BMI (kg/m^2^)	29.44 ± 5.94	28.65 ± 5.48	29.97 ± 6.18	0.000	30.29 ± 5.44	21.35 ± 4.12	0.000
VAI	3.10 ± 2.66	3 ± 2.88	3.17 ± 2.50	0.175	3.28 ± 2.72	1.37 ± 0.81	0.000
LAP	76.65 ± 69.29	84.11 ± 87.87	71.63 ± 52.74	0.000	83.12 ± 69.68	15.31 ± 1.30	0.000
ABSI	0.81 ± 0.05	0.81 ± 0.05	0.80 ± 0.05	0.000	0.81 ± 0.05	0.76 ± 0.06	0.000
BRI	5.83 ± 2.13	5.38 ± 1.90	6.14 ± 2.22	0.000	6.18 ± 1.94	2.57 ± 0.50	0.000
DAAT	187.85 ± 82.03	233.22 ± 89.80	157.37 ± 59.35	0.000	199.80 ± 75.99	74.62 ± 40.03	0.000
WWI	11.28 ± 0.90	11.08 ± 0.83	11.42 ± 0.91	0.000	11.42 ± 0.78	9.92 ± 0.74	0.000
HOMA-IR(mg/dL)	3.41 ± 5.05	3.12 ± 5.05	3.61 ± 5.04	0.038	3.63 ± 5.24	1.14 ± 1.62	0.000
TG (mg/dL)	8.88 ± 0.61	8.98 ± 0.65	8.81 ± 0.57	0.000	8.94 ± 0.59	8.31 ± 0.46	0.000
TG*BMI	262.59 ± 59.96	258.78 ± 58.49	265.15 ± 60.82	0.024	271.54 ± 54.67	177.79 ± 37.37	0.000
TG*WC	862.84 ± 152.66	888.45 ± 160	845.24 ± 145.25	0.000	888.27 ± 135.89	619.25 ± 68.40	0.000
TG/HDL(mmol)	1.87 ± 1.66	2.22 ± 2.08	1.63 ± 1.24	0.000	1.97 ± 1.70	0.94 ± 0.57	0.000
QUICKI(mUI)	2.22 ± 0.08	2.23 ± 0.09	2.22 ± 0.08	0.044	2.23 ± 0.09	2.21 ± 0.06	0.002
AIP	0.17 ± 0.28	0.23 ± 0.29	0.13 ± 0.26	0.000	0.20 ± 0.27	0.08 ± 0.22	0.000
Glucose (mg/dL)	97.65 ± 33.92	97.84 ± 31.62	97.52 ± 35.40	0.837	98.61 ± 35.02	88.53 ± 18.50	0.000
Cholesterol (mg/dL)	178.65 ± 57.27	182.19 ± 78.22	176.26 ± 36.90	0.028	181.16 ± 58.60	154.79 ± 34.52	0.000
Triglycerides (mg/dL)	178.93 ± 127.19	204.72 ± 160.89	161.60 ± 94.46	0.000	186.91 ± 130.16	103.30 ± 51.32	0.000
LDL-C (mg/dL)	107.33 ± 28.99	108.65 ± 29.72	106.45 ± 28.48	0.106	109.22 ± 28.48	89.43 ± 27.76	0.000
HDL-C (mg/dL)	45.55 ± 10.09	43.69 ± 9.75	46.80 ± 10.12	0.000	44.97 ± 9.81	50.98 ± 11.04	0.000
Uric acid (mg/dL)	5.14 ± 1.43	6.07 ± 1.39	4.52 ± 1.08	0.000	5.20 ± 1.43	4.59 ± 1.30	0.000
Albumin (g/dL)	4.20 ± 0.30	4.29 ± 0.32	4.13 ± 0.28	0.000	4.18 ± 0.30	4.35 ± 0.31	0.000
Creatinine mg/dL	0.73 ± 0.33	0.88 ± 0.46	0.64 ± 0.13	0.000	0.73 ± 0.33	0.77 ± 0.29	0.112
Insulin UI/mL	13.48 ± 15.50	12.20 ± 14.86	14.34 ± 15.86	0.003	14.23 ± 15.98	6.39 ± 6.50	0.000
CRP (mg/L)	0.43 ± 0.85	0.39 ± 0.96	0.46 ± 0.78	0.061	0.45 ± 0.80	0.28 ± 1.25	0.014
HBA1c (%)	5.58 ± 0.86	5.59 ± 0.95	5.57 ± 0.80	0.743	5.62 ± 0.88	5.21 ± 0.55	0.00
Systolic (mmHg)	119.60 ± 17.77	125.15 ± 16.75	115.86 ± 17.46	0.000	120.46 ± 17.84	111.35 ± 14.76	0.000
Diastolic (mmHg)	74.90 ± 11.34	76.84 ± 11.49	73.60 ± 11.06	0.000	75.56 ± 11.29	68.62 ± 9.87	0.000

WC, Waist circumference; WHtR, waist-to-height ratio; BMI, body mass index; VAI, visceral adiposity index; LAP, lipid accumulation product; ABSI, body shape index; BRI, body roundness index; DAAT, Deep abdominal adipose tissue; WWI, weight-adjusted waist index; HOMA-IR, Homeostasis Model Assessment insulin resistance; TG, Triglycerides-Glucose Index; TG/HDL-C, Triglycerides to high-density lipoprotein cholesterol ratio; QUICKI, Quantitative insulin sensitivity check index; AIP, Atherogenic index of plasma; LDL-C, low-density lipoprotein cholesterol; HDL-C, high-density lipoprotein cholesterol; CRP, C-reactive protein; HBA1c, Glycated Hemoglobin; TG*BMI, Triglycerides-Glucose index multiplied by body mass index; TG*WC, Triglycerides-Glucose Index multiplied by waist circumference.

Compared to the non-CMR group, CMR participants exhibited significantly elevated values for all anthropometric and biochemical parameters (p<0.001 for all variables). The CMR group demonstrated markedly higher mean BMI (30.39 vs. 25.79 kg/m²), waist circumference (105.53 vs. 83.92 cm), and WHtR (0.64 vs. 0.50). Advanced adiposity indices showed pronounced differences, with BRI (6.82 vs. 4.13), DAAT (215.26 vs. 142.64), and LAP (67.29 vs. 26.12) being substantially elevated in the CMR group. Biochemical profiles revealed significant metabolic dysfunction in CMR participants, with higher fasting glucose (104.18 vs. 92.41 mg/dL), insulin (14.01 vs. 10.74 μIU/mL), and HOMA-IR (3.64 vs. 2.45). Lipid parameters demonstrated atherogenic patterns in the CMR group, including elevated triglycerides (192.44 vs. 138.21 mg/dL), reduced HDL-C (44.95 vs. 46.84 mg/dL), and higher TG/HDL ratio (1.89 vs. 1.23). Combined indices TGBMI (284.65 vs. 178.23) and TGWC (945.19 vs. 582.61) were markedly elevated in CMR participants.

Sex-Specific Patterns Within CMR Categories.

Among participants with CMR, significant sex differences persisted. Males exhibited higher visceral adiposity markers including DAAT (245.63 vs. 195.73, p<0.001), WWI (11.59 vs. 11.44, p<0.001), and ABSI (0.082 vs. 0.081, p<0.001). Biochemical profiles showed males with more severe dyslipidemia, evidenced by higher triglycerides (219.56 vs. 174.52 mg/dL, p<0.001), TG/HDL ratio (2.22 vs. 1.67, p<0.001), and AIP (0.28 vs. 0.15, p<0.001). Conversely, females with CMR demonstrated higher subcutaneous adiposity patterns with elevated BMI (31.18 vs. 29.18 kg/m², p<0.001), WHtR (0.66 vs. 0.61, p<0.001), and BRI (7.24 vs. 5.94, p<0.001), alongside higher insulin levels (15.28 vs. 11.96 μIU/mL, p<0.001) and HOMA-IR (3.95 vs. 3.17, p<0.001).

In the non-CMR group, sex differences were less pronounced but still significant. Males maintained higher waist circumference (89.24 vs. 83.54 cm, p<0.001) and triglycerides (151.98 vs. 129.89 mg/dL, p<0.001), while females showed marginally higher BMI (26.05 vs. 25.30 kg/m², p=0.012) and insulin sensitivity indices.

### 3.1 Partial correlation between different anthropometric and biochemical indexes

The anthropometric indices exhibited positive correlations with one another, with WHtR, BRI, and WWI demonstrating the strongest correlations, followed by BMI and DAAT. The ABSI showed correlation values of < 0.500 (p<0.001) (**Table 2**).

**Table 2:** Pearson’s correlation test for anthropometric indices.

Index	Pearson	BMI	WHtR	ABSI	BRI	DAAT	WWI
BMI	Correlation Sig.	1	0.846** 0.000	-0.107** 0.000	0.851** 0.000	0.664** 0.000	0.342** 0.000
WHtR	Correlation Sig.	0.846** 0.000	1	0.384** 0.000	0.993** 0.000	0.722** 0.000	0.783** 0.000
ABSI	Correlation Sig.	-0.107** 0.000	0.384** 0.000	1	0.365** 0.000	0.423** 0.000	0.816** 0.000
BRI	Correlation Sig.	0.851** 0.000	0.993** 0.000	0.365** 0.000	1	0.710** 0.000	0.765** 0.000
DAAT	Correlation Sig.	0.664** 0.000	0.722** 0.000	0.423** 0.000	0.710** 0.000	1	0.495** 0.000
WWI	Correlation Sig.	0.342** 0.000	0.783** 0.000	0.816** 0.000	0.765** 0.000	0.495** 0.000	1

**Correlation is significant at the 0.01 level (bilateral).

*Correlation is significant at p < 0.05 (bilateral).

WHtR, waist-to-height ratio; BMI, body mass index; ABSI, body shape index; BRI, body roundness index; DAAT, Deep abdominal adipose tissue; WWI, weight-adjusted waist index.

The biochemical indices were contrasted with Pearson’s correlation statistic, considering strong correlations with a higher value of (Pearson 0.500) and (*p<0.001*). The best indices with a higher correlation were TG, TG/HDL, and AIP (*p<0.001*) than the HOMA-IR and QUICKI indices, both of which had low correlations (**Table 3**).

**Table 3:** Pearson’s correlation test for biochemical indices.

Index	Pearson	HOMA-IR	TG	TG/HDL	QUICKI	AIP
HOMA-IR	Correlation Sig.	1	0.291** 0.000	0.157** 0.000	0.248** 0.000	0.195** 0.000**
TG	Correlation Sig.	0.291** 0.000	1	0.779** 0.000	0.408** 0.000	0.898** 0.000
TG/HDL	Correlation Sig.	0.157** 0.000	0.779** 0.000	1	0.108** 0.000	0.841** 0.000
QUICKI	Correlation Sig.	0.248** 0.000	0.408** 0.000	0.108** 0.000	1	0.087** 0.000
AIP	Correlation Sig.	0.195** 0.000	0.898** 0.000	0.841** 0.000	0.087** 0.000	1

**Correlation is significant at the 0.01 level (bilateral). HOMA-IR Homeostasis Model Assessment insulin resistance; TG, Triglycerides-Glucose Index; TG/HDL-C, Triglycerides to high-density lipoprotein cholesterol ratio; QUICKI, Quantitative insulin sensitivity check index; AIP, Atherogenic index of plasma.

Regarding the correlations between the indices by sex, we observed that in both sexes, the indices with the highest correlation were WHtR, BRI, and DAAT, as they demonstrated a correlation with LAP, TG*BMI, and TG*WC indices (p<0.001). The most effective female indicators were BRI and DAAT, which exhibited higher correlation values, whereas ABSI did not demonstrate higher correlations. Irrespective of the correlation value, anthropometric indicators exhibited a relationship with most of the biochemical indicators. In men, QUICKI had the lowest correlation with the other indicators, as it correlated only with ABSI and WWI. Conversely, although the correlation value was low in women, a correlation was observed for all anthropometric indices. The ABSI demonstrated weak correlations, potentially due to the formula’s utilization of two anthropometric measurements (weight and height) and one index (BMI), which may have led to variations in the results. Specific indicators can be employed in conjunction regardless of sex, and our findings indicate that anthropometric indicators behave similarly irrespective of sex. However, biochemical indicators, such as QUICKI, yield more favorable results for females.

### 3.2 Diagnostic ability of anthropometric and biochemical indicators

**Figure 1:**
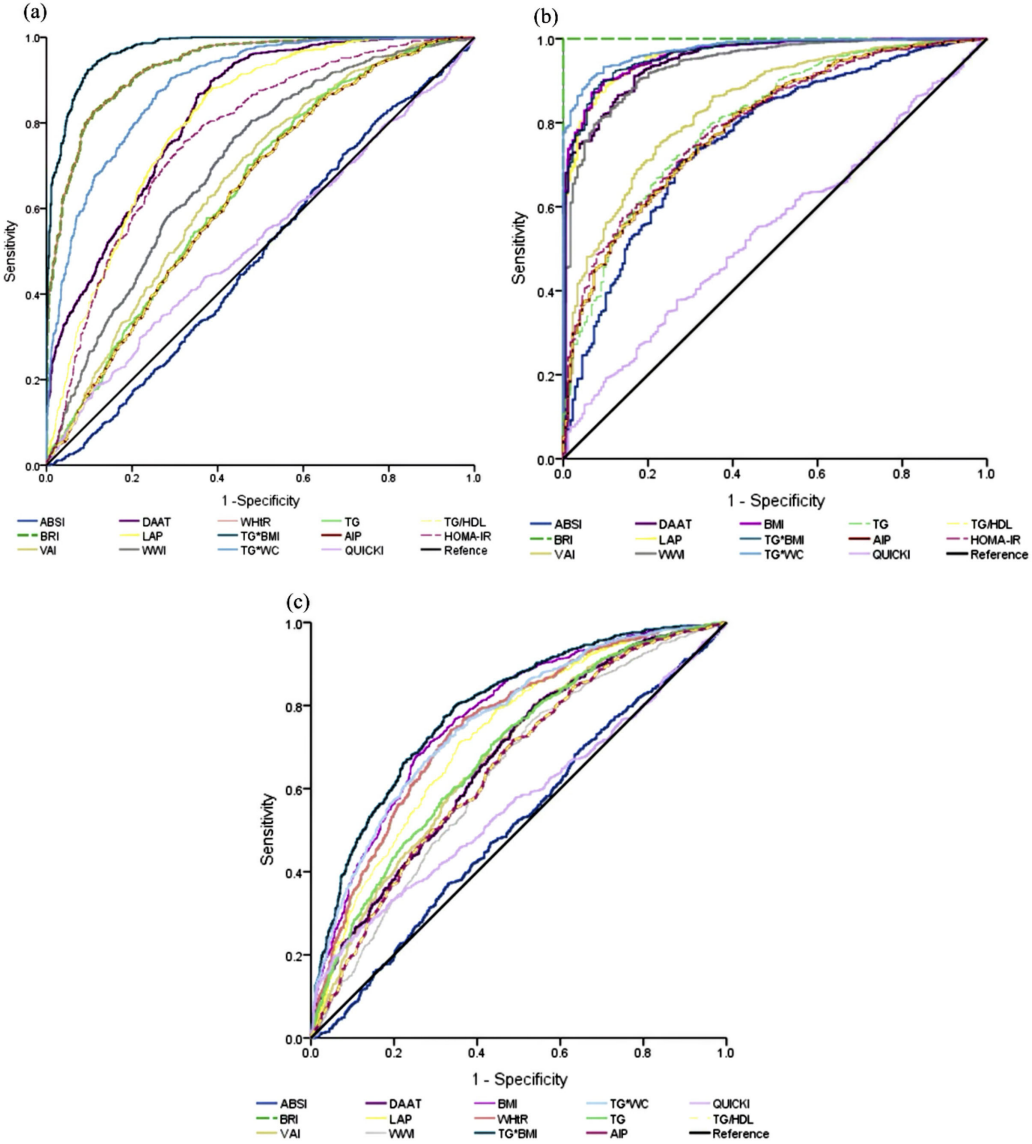
Receiver operating characteristic (ROC) curve **(a)** illustrates the ROC curve with anthropometric indices using BMI as the contrast variable. **(b)** depicts the anthropometric and biochemical indices with the WHtR serving as the contrast variable, whereas **(c)** presents the biochemical indicators with the HOMA-IR index as the contrast variable. The analysis was conducted for the entire study population.

To evaluate the diagnostic performance of the calculated anthropometric indices in obesity assessment, we used Receiver Operating Characteristic (ROC) curve analysis, with Body Mass Index (BMI) employed as the reference standard for obesity classification. The ROC analysis revealed that TG*BMI (AUC=0.972), followed by WHtR (AUC=0.934), BRI (AUC=0.934), TG*WC (0.888), DAAT (0.825), and LAP (0.805), demonstrated higher area under the curve (AUC) values. Conversely, HOMA-IR (0.769), WWI (0.705), and VAI (0.664) (p<0.005) exhibited lower values for determining adiposity, whereas ABSI (0.490) and QUICKI (0.525) showed no useful discriminative ability. Although ABSI does not contribute to adiposity determination, it helps assess body shape (**Figure 1a**).

The analysis of the indicators’ ability to assess CMR, utilizing the WHtR index as a contrast indicator, revealed that the indicators with higher AUC values were BRI (AUC=1.000), followed by TG*WC (0.976), LAP (0.963), BMI (0.961), TG*BMI (0.960), DAAT (0.953), WWI (0.940) the VAI (0.840), TG (0.803), HOMA-IR (0.799), TG/HDL (0.794) and AIP (0.794), ABSI (0.763) (p<0.005), indices exhibited moderate predictive capacity and QUICKI (0.548) which demonstrated the lowest efficacy in assessing CMR (**Figure 1b**).

The HOMA-IR index was used to evaluate the capacity to assess insulin resistance. The findings indicated that The TG*BMI (0.792), TG*WC (0.761), BMI (0.772), LAP (0.726), WHtR (0.749), and BRI (0.749) indices exhibited moderate AUC values, whereas the indices that demonstrated lower values were QUICKI (0.564), AIP (0.652), TG/HDL (0.652), VAI (0.677), DAAT (0.674), WWI (0.635), and TG (0.687) (p<0.005) (**Figure 1c**).

### 3.3 Differences in ROC curves of anthropometric and biochemical indices by sex

**Figure 2:**
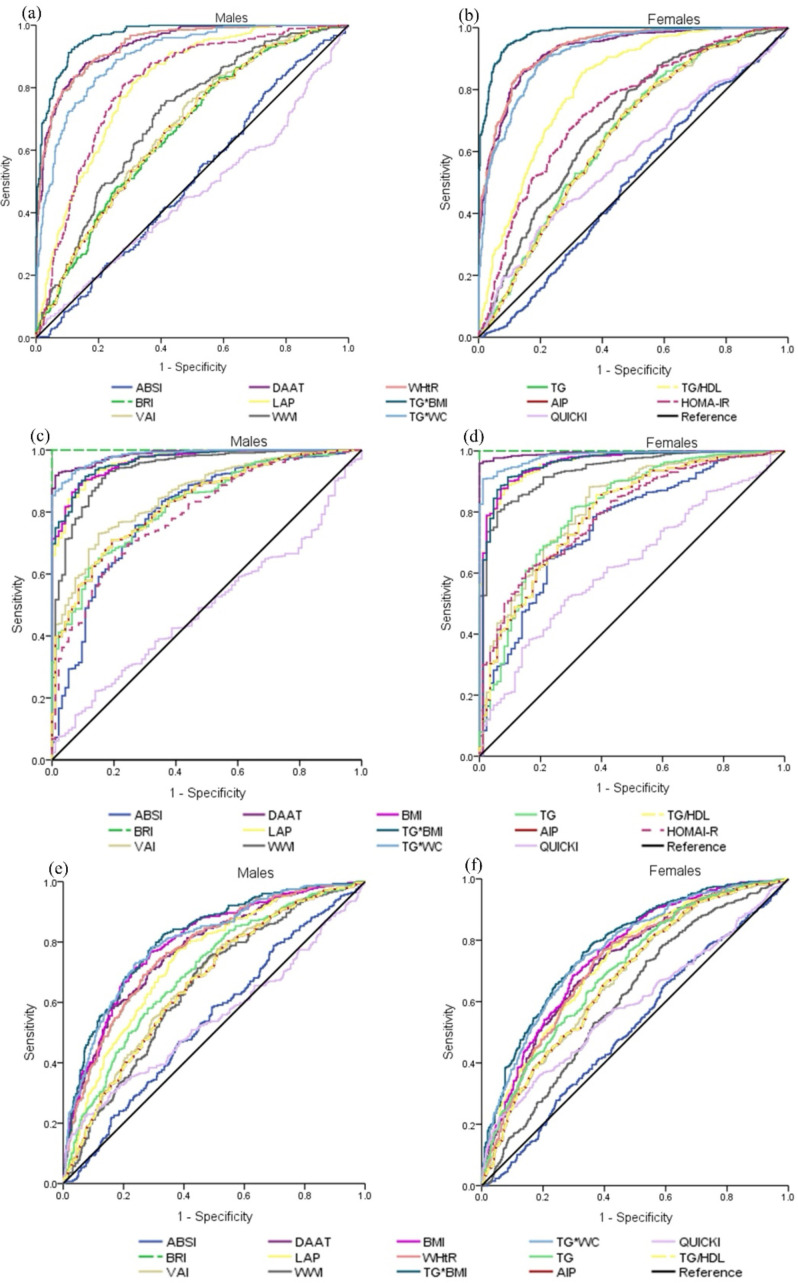
Receiver operating characteristic (ROC) curves according to sex. **(a, b)** illustrate the obesity indicators, with the BMI index utilized as a contrast variable; **(c, d)** depict the RCM indicators, with the WHtR index employed as a contrast variable; **(e, f)** present the insulin resistance indicators, with the HOMA-IR index serving as a contrast variable. The population was stratified by sex for all the analyses.

The study revealed that in both male and female participants, the indicators demonstrating superior adiposity evaluation capability by AUC were TG*BMI (AUC 0.965 and 0.978, male and female, respectively), WHtR (0.936, 0.932), BRI (0.936, 0.932), DAAT (0.935, 0.926), TG*WC (0.901, 0.915), and LAP (0.811, 0.813). In men, the HOMA-IR index demonstrated a strong predictive value for obesity (0.809) (p<0.005) (**Figures 2a, b**). In assessing the ability to evaluate CMR, more indicators exhibited superior performance in both sexes: BRI (1.000, 1.000), DAAT (0.986, 0.994), TG*WC (0.984, 0.984), TG*BMI (0.967, 0.956), BMI (0.965, 0.957), WWI (0.939, 0.938), TG (0.823, 0.808), and VAI (0.854, 0.819). In men, the AIP (0.827), TG/DHL (0.827), and ABSI (0.800) indices were better predictors of CMR, whereas in women, these predictors were not superior (p<0.005) (**Figures 2c, d**).

To detect insulin resistance, the indicators that demonstrated superiority in men were TG*BMI (0.812) and TG*WC (0.800); in women, the values were lower in these indices TG*BMI (0.779) and TG*WC (0.767) (p<0.005); other indices that were moderately predictive of IR were BMI (0.795, 0.751), DAAT (0.775, 0.727), WHtR (0.773, 0.724), and BRI (0.773, 0.724) (**Figures 2e, f**). Based on these observations, we suggest that these indices may be applied to the general population, irrespective of sex. The study also identified indices that are not recommended for use owing to their low values, namely, TG/DHL, AIP, and QUICKI.

### 3.4 Subgroup analysis

#### 3.4.1 Visceral adiposity indices performance

**Figure 3:**
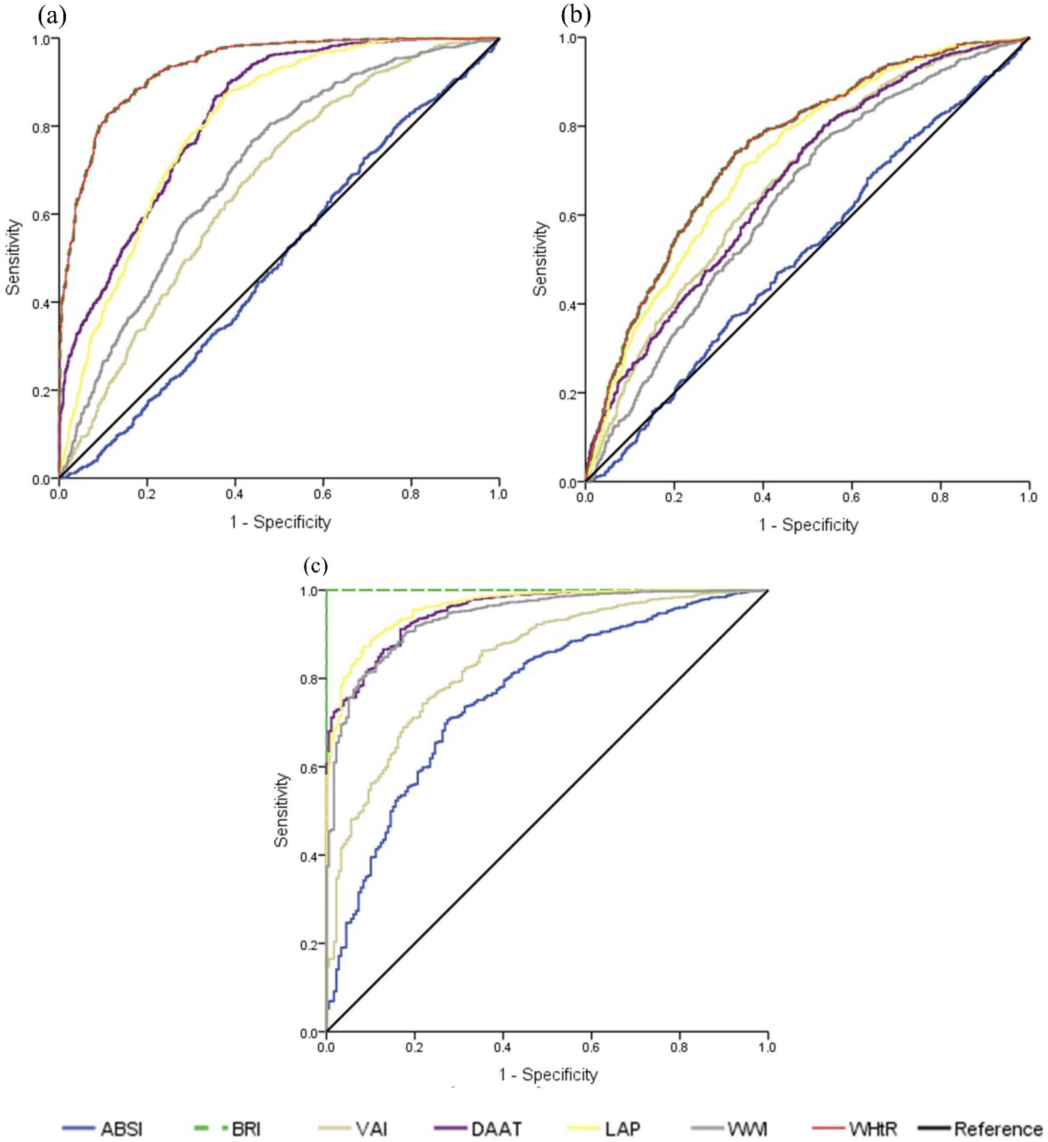
receiver operating characteristic (ROC) curves based on indices related to visceral adipose tissue. **(a)** illustrates the obesity indicators using body mass index (BMI) as the reference variable. **(b)** shows the homeostatic model assessment of insulin resistance (HOMA-IR) index serving as the reference variable. **(c)** depicts the cardiometabolic risk (CMR) indicators, with the waist-to-height ratio (WHtR) as the reference variable. The analysis was conducted on the total population.

To address potential interactions and subgroup effects, we conducted targeted ROC analyses focusing on indices specifically designed to assess visceral adipose tissue dysfunction and cardiometabolic risk factors (**Figure 3**).

Using BMI as the reference standard, BRI and WHtR demonstrated excellent discriminatory capacity (AUC=0.934, p<0.001), indicating a superior ability to identify obesity compared to other visceral adiposity markers. DAAT showed good performance (AUC=0.825), whereas LAP achieved acceptable discrimination (AUC=0.805). WWI demonstrated moderate utility (AUC=0.705), suggesting its potential as a complementary assessment tool. Notably, VAI showed limited obesity discrimination (AUC=0.664), reflecting its design as a metabolic rather than a purely anthropometric indicator. ABSI performed poorly for obesity detection (AUC=0.490, p=0.478), which is consistent with its primary utility for mortality prediction rather than adiposity assessment **Figure 3a**.

For insulin resistance discrimination using HOMA-IR as reference, BRI, WHtR, and LAP achieved comparable superior performance (AUC=0.749, p<0.001). This finding underscores the strong relationship between central adiposity and insulin resistance pathophysiology. VAI, DAAT, and WWI demonstrated moderate discriminatory ability (AUC=0.635-0.677), while ABSI showed inadequate performance (AUC=0.513, p=0.327) for insulin resistance detection in this population **Figure 3b**.

For cardiometabolic risk, using WHtR ≥0.50 as the CMR reference, LAP demonstrated exceptional discrimination (AUC=0.963), followed closely by DAAT (AUC=0.953), reflecting the superior performance of combined anthropometric-biochemical indices. WWI achieved excellent discrimination (AUC=0.940), whereas VAI showed good performance (AUC=0.840). Interestingly, ABSI demonstrated acceptable CMR discrimination (AUC=0.763), suggesting differential utility across cardiometabolic outcomes **Figure 3c**.

#### 3.4.2 Combined biochemical-anthropometric index performance

**Figure 4:**
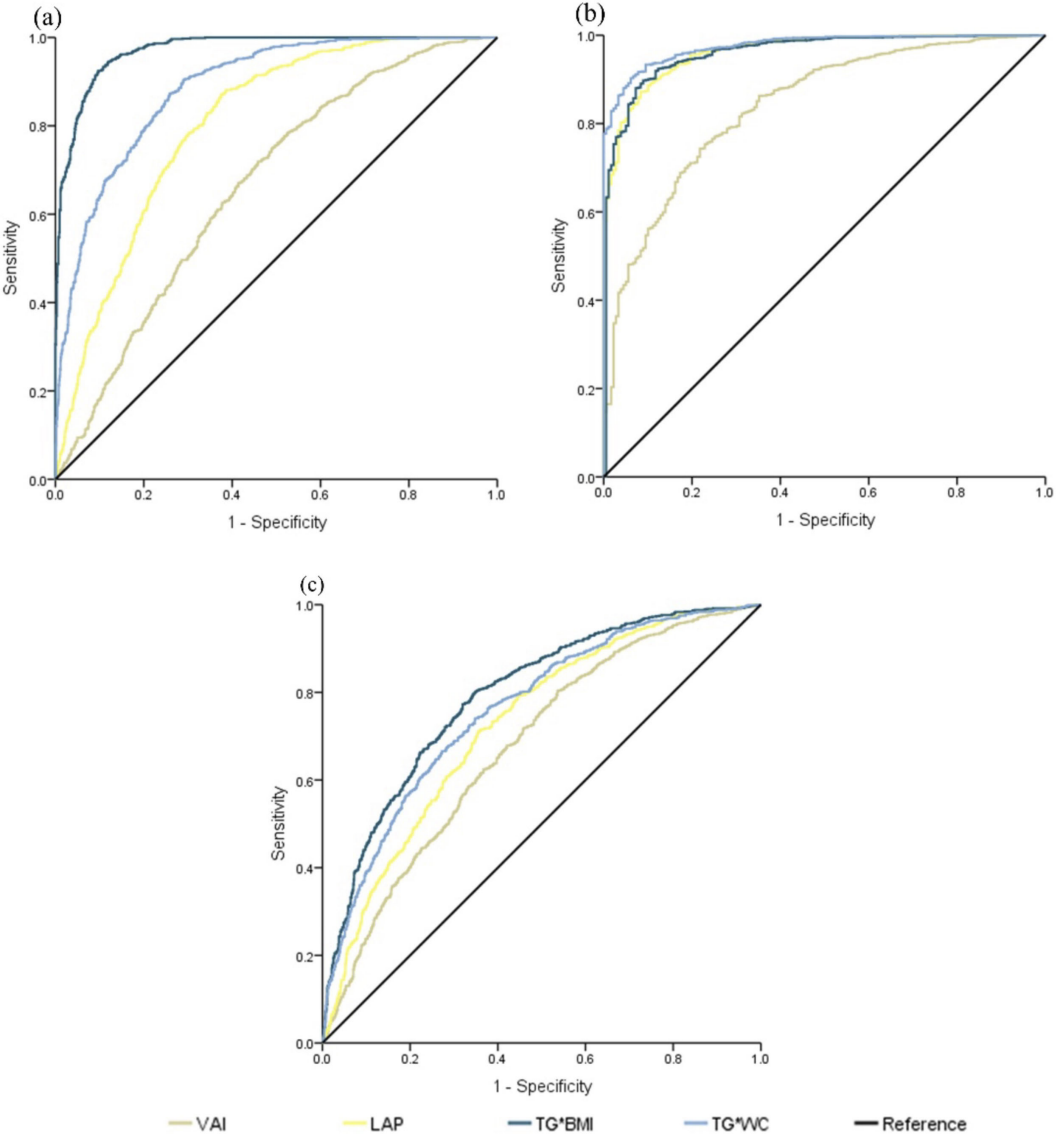
Receiver operating characteristic (ROC) curves based on biochemical indices with anthropometric measurements. **(a)** illustrates the obesity indicators using body mass index (BMI) as the reference variable. **(b)** depicts the cardiometabolic risk (CMR) indicators with the waist-to-height ratio (WHtR) as the reference variable. **(c)** shows the homeostatic model assessment of insulin resistance (HOMA-IR) index serving as the reference variable. The analysis was conducted on the total population.

We conducted comprehensive subgroup analyses using combined biochemical-anthropometric indices to address potential interactions and enhance diagnostic precision (**Figure 4**). For obesity prediction using combined indices, ROC analysis of biochemical-anthropometric combinations revealed superior performance compared to individual measures. TG*BMI demonstrated exceptional discriminatory capacity for obesity detection (AUC=0.972, 95% CI: 0.966-0.978), significantly outperforming individual biochemical indices. TG*WC achieved good discrimination (0.888), whereas LAP showed acceptable performance (0.805). The VAI demonstrated limited utility for obesity assessment (0.664), reflecting its primary design for metabolic dysfunction rather than for adiposity quantification (**Figure 4a**).

For CMR prediction, TG*WC achieved near-perfect discrimination (AUC=0.976, 95% CI: 0.971-0.981), likely attributable to the incorporation of waist circumference—a key component in our WHtR-based CMR classification. This finding supports the biological plausibility that waist-circumference-based indices effectively capture central adiposity patterns associated with cardiometabolic dysfunction. TG*BMI maintained excellent performance (AUC=0.945), whereas LAP and VAI showed good discriminatory capacities (AUC=0.869 and 0.840, respectively) (**Figure 4b**).

Combined indices demonstrated moderate-to-good performance for insulin resistance assessment, with TG*BMI achieving the highest discrimination (AUC=0.792, 95% CI: 0.772-0.812). TGWC and LAP showed comparable performance (AUC=0.761 and 0.726, respectively), whereas VAI exhibited limited utility (AUC=0.677, p<0.005). The moderate performance across all indices suggests that insulin resistance assessment may require alternative biomarkers or multiparameter approaches in this population (**Figure 4c**).

Our findings demonstrate that combining triglyceride levels with anthropometric measures significantly enhances the diagnostic accuracy across cardiometabolic outcomes. The superior performance of TG*BMI and TG*WC supports their potential as practical screening tools, particularly in resource-limited settings where comprehensive metabolic panels may be unavailable.

### 3.4 The optimal cut-off value of anthropometric indices

#### 3.4.1 Obesity prediction

ROC analysis revealed that TG*BMI demonstrated the highest diagnostic performance for obesity detection (AUC=0.972, 95% CI: 0.966-0.978), followed by WHtR and BRI (both AUC=0.934), and TG*WC (AUC=0.898). The optimal cut-off values were 266.97 for TGBMI, 0.62 for WHtR, 5.05 for BRI, and 461.73 for TGWC. These indices achieved high sensitivity (0.825-0.905) and specificity (0.888-0.903), with Youden indices ranging from 0.71-0.80, indicating excellent discriminatory capacity **Table 4**.

**Table 4:** The optimal cut-off value of anthropometric indices.

	AUC (95%CI)	Cut-off value	Sensitivity	Specificity	Youden index
Obesity					
TG*BMI	0.972 (0.966-0.978)**	266.97	0.925	0.903	0.827
WHtR	0.934 (0.924-0.945)**	0.62	0.829	0.888	0.717
BRI	0.934 (0.924-0.945)**	6.05	0.829	0.888	0.717
TG*WC	0.888 (0.874-0.902)**	840.73	0.905	0.710	0.614
DAAT	0.825 (0.807-0.843)**	161.63	0.897	0.616	0.513
LAP	0.805 (0.786-0.824)**	51.64	0.870	0.625	0.496
HOMA-IR	0.769 (0.748-0.791)**	2.12	0.775	0.650	0.425
WWI	0.705 (0.682-0.728)**	11.12	0.765	0.564	0.329
VAI	0.664 (0.640-0.688)**	2.17	0.719	0.541	0.260
Cardiometabolic risk					
BRI	1.000 (1,000–1,000)**	3.27	1.000	1.000	1.000
TG*WC	0.976 (0.969-0.983)**	722.41	0.903	0.933	0.836
LAP	0.963 (0.951-0.974)**	30.75	0.872	0.916	0.788
BMI	0.961 (0.946-0.975)**	24.27	0.902	0.905	0.807
TG*BMI	0.960 (0.946-0.975)**	208.68	0.897	0.911	0.808
DAAT	0.953 (0.941-0.966)**	109.54	0.912	0.832	0.744
WWI	0.940 (0.924-0.956)**	10.46	0.904	0.821	0.725
VAI	0.840 (0.811-0.870)**	1.78	0.743	0.782	0.525
TG	0.803 (0.769-0.836)**	8.60	0.711	0.743	0.454
HOMA-IR	0.799 (0.767-0.831)**	1.58	0.727	0.715	0.442
AIP	0.794 (0.761-0.827)**	0.017	0.745	0.682	0.427
TG/HDL	0.794 (0.761-0.827)**	1.04	0.745	0.682	0.427
ABSI	0.763 (0.726-0.800)**	0.790	0.706	0.726	0.431
Insuline resistence					
TG*BMI	0.792 (0.772-0.812)**	251.37	0.798	0.654	0.453
BMI	0.772 (0.751-0.793)**	29.28	0.712	0.713	0.425
TG*WC	0.761 (0.739-0.782)**	881.99	0.665	0.728	0.393
WHtR	0.749 (0.727-0.771)**	0.60	0.741	0.663	0.404
BRI	0.749 (0.727-0.771)**	5.54	0.741	0.663	0.404
LAP	0.726 (0.704-0.749)**	59.62	0.714	0.640	0.354

**p value <0.005, TG*BMI, Triglycerides-Glucose index multiplied by body mass index; WHtR, waist-to-height ratio; BRI, body roundness index; TG*WC, Triglycerides-Glucose Index multiplied by waist circumference; DAAT, Deep abdominal adipose tissue; LAP, lipid accumulation product; BMI, body mass index; TG, Triglycerides-Glucose Index; HOMA-IR, Homeostasis Model Assessment insulin resistance; WWI, weight-adjusted waist index; VAI, visceral adiposity index; AIP, Atherogenic index of plasma; TG/HDL-C, Triglycerides to high-density lipoprotein cholesterol ratio; ABSI, body shape index.

Sex-stratified analysis revealed differential diagnostic performance; in males, TG*BMI demonstrated superior obesity prediction (AUC=0.965, 95% CI: 0.954-0.976) with an optimal cut-off of 268.39, achieving high sensitivity (0.923) and specificity (0.886). WHtR, BRI, and DAAT showed comparable performance (AUC=0.932-0.936) with cut-offs of 0.61, 5.76, and 246.56, respectively. TGWC exhibited a good discriminatory capacity (AUC=0.902), with a cut-off of 915.38 **Table 5**.

**Table 5:** Optimal cut-off values of anthropometric indices according to sex for detecting obesity, cardiometabolic risk, and insulin resistance.

	AUC (95%CI)	Cut-off	Sensitivity	Specificity	Youden index
Obesity					
Male					
TG*BMI	0.965 (0.954-0.976)	268.39	0.923	0.886	0.809
WHtR	0.936 (0.919-0.953)	0.61	0.835	0.870	0.715
BRI	0.936 (0.919-0.953)	5.76	0.835	0.879	0.715
DAAT	0.935 (0.918-0.952)	246.56	0.875	0.844	0.720
TG*WC	0.902 (0.879-0.922)	915.38	0.832	0.811	0.642
LAP	0.811 (0.781-0.841)	64.77	0.813	0.692	0.505
HOMA-IR	0.809 (0.777-0.840)	2.06	0.806	0.723	0.529
Female					
TG*BMI	0.978 (0.972-0.985)	263.45	0.942	0.908	0.850
WHtR	0.932 (0.918.-0.946)	0.63	0.847	0.872	0.719
BRI	0.932 (0.918-0.946)	6.17	0.847	0.872	0.719
DAAT	0.926 (0.911-0.941)	159.77	0.858	0.855	0.713
TG*WC	0.915 (0.899-0.931)	838.74	0.879	0.809	0.688
LAP	0.813 (0.788-0.838)	51.64	0.854	0.666	0.520
HOMA-IR	0.738 (0.709-0.766)	2.41	0.718	0.663	0.380
Cardiometabolic risk					
Male					
BRI	1.000 (1.000-1.000)	3.29	1.000	1.000	1.000
DAAT	0.986 (0.979-0.993)	159.11	0.918	0.989	0.908
TG*WC	0.984 (0.976-0.992)	759.57	0.894	0.868	0.862
TG*BMI	0.967 (0.953-0.981)	208.70	0.915	0.892	0.808
BMI	0.965 (0.951-0.980)	24.27	0.902	0.903	0.805
WWI	0.939 (0.914-0.964)	10.34	0.923	0.828	0.751
VAI	0.854 (0.817-0.891)	1.75	0.731	0.849	0.580
TG/DHL	0.827 (0.787-0.867)	1.33	0.710	0.806	0.516
TG	0.823 (0.783-0.863)	8.83	0.619	0.882	0.500
ABSI	0.800 (0.750-0.851)	0.796	0.756	0.731	0.448
Female					
BRI	1.000 (1.000-1.000)	3.26	1.000	1.000	1.00
DAAT	0.994 (0.990-0.997)	88.58	0.959	1.000	0.959
TG*WC	0.984 (0.976-0.991)	700.91	0.908	0.988	0.897
TG*BMI	0.956 (0.931-0.982)	203.23	0.914	0.907	0.821
BMI	0.957 (0.931-0.981)	24.27	0.902	0.907	0.809
WWI	0.938 (0.917-0.959)	10.80	0.801	0.942	0.743
VAI	0.819 (0.772- 0.866)	1.38	0.882	0.640	0.522
TG/DHL	0.794 (0.743-0.844)	0.77	0.856	0.616	0.472
TG	0.808 (0.756-0.860)	8.38	0.813	0.698	0.510
ABSI	0.753 (0.700-0.806)	0.78	0.648	0.779	0.427
Insulin resistance					
Male					
TG*BMI	0.812 (0.781-0.843)	252.27	0.824	0.669	0.494
TG*WC	0.800 (0.768-0.833)	915.38	0.731	0.760	0.491
BMI	0.795 (0.762-0.828)	28.65	0.767	0.712	0.479
DAAT	0.775 (0.741-0.809)	270.60	0.573	0.848	0.422
WHtR	0.773 (0.739-0.807)	0.59	0.735	0.691	0.425
BRI	0.773 (0.739-0.807)	5.37	0.735	0.691	0.425
LAP	0.738(0.703-0.774)	59.71	0.774	0.619	0.393
Female					
TG*BMI	0.779 (0.753-0.806)	256.09	0.760	0.670	0.430
TG*WC	0.767 (0.739-0.794)	842.95	0.719	0.699	0.417
BMI	0.751 (0.723-0.779)	29.75	0.686	0.700	0.387
DAAT	0.727 (0.698-0.756)	148.52	0.740	0.613	0.354
WHtR	0.724 (0.695-0.754)	0.61	7.38	0.635	0.373
BRI	0.724 (0.695-0.754)	5.70	0.738	0.635	0.373
LAP	0.729 (0.700-0.758)	50.13	0.774	0.580	0.354

TG*BMI, Triglycerides-Glucose index multiplied by body mass index; WHtR, waist-to-height ratio; BRI, body roundness index; TG*WC, Triglycerides-Glucose Index multiplied by waist circumference; DAAT, Deep abdominal adipose tissue; LAP, lipid accumulation product; BMI, body mass index; TG, Triglycerides-Glucose Index; HOMA-IR, Homeostasis Model Assessment insulin resistance; WWI, weight-adjusted waist index; VAI, visceral adiposity index; TG/HDL-C, Triglycerides to high-density lipoprotein cholesterol ratio; ABSI, body shape index.

In females, BRI achieved perfect diagnostic accuracy (AUC=1.000, 95% CI: 1.000-1.000) with a cut-off of 6.17, demonstrating 100% sensitivity and specificity. WHtR maintained a strong performance (AUC=0.932) with a cut-off of 0.63, whereas TG*BMI showed excellent discrimination (AUC=0.984) at a cut-off of 263.45. Notably, female-specific cut-offs were generally higher for most indices than for males, reflecting sex-specific adiposity patterns **Table 5**.

#### 3.4.2 Cardiometabolic risk assessment

BRI exhibited perfect diagnostic performance for CMR detection (AUC=1.000, 95% CI: 1.000-1.000) with an optimal cut-off of 3.27, achieving 100% sensitivity and specificity (Youden index=1.00). TG*WC demonstrated exceptional performance (AUC=0.975, 95% CI: 0.969-0.980) with a cut-off of 722.41, followed by LAP (AUC=0.969, cut-off=30.75). Notably, 13 indices achieved AUC values >0.75, indicating good-to-excellent diagnostic accuracy for CMR identification. Traditional BMI showed moderate performance (AUC=0.858) with an optimal cut-off of 27.24 kg/m² **Table 4**.

For CMR prediction, although both sexes demonstrated exceptional performance with BRI achieving perfect discrimination, cut-offs were different: 3.23 (males) and 3.26 (females). DAAT showed excellent performance in both groups (male AUC=0.986, cut-off=159.11; female AUC=0.984, cut-off=89.58). TG*WC demonstrated strong predictive capacity with sex-specific cut-offs of 759.67 (males) and 700.91 (females), both achieving AUC values >0.980 **Table 5**.

Traditional BMI maintained good performance across sexes (male AUC=0.965, female AUC=0.957) with similar cut-offs (24.27 vs. 24.27 kg/m²). Additional indices, including WWI, VAI, TG/HDL, TG, and ABSI, showed consistent performance between sexes, indicating robust CMR detection capabilities, regardless of sex-specific metabolic differences **Table 5**.

#### 3.4.3 Insulin resistance detection

For insulin resistance assessment, TG*BMI achieved the highest discriminatory power (AUC=0.792, 95% CI: 0.772-0.812) with a cut-off of 251.37, followed by BMI (AUC=0.772, cut-off=29.28 kg/m²) and TG*WC (AUC=0.761, cut-off=861.99). However, overall diagnostic performance for insulin resistance was lower compared to obesity and CMR detection, with most indices achieving moderate accuracy (AUC 0.70-0.80) **Table 4**.

Sex differences were most pronounced in insulin resistance assessment. Males showed superior diagnostic performance with TG*BMI (AUC=0.812, cut-off=252.27) and TG*WC (AUC=0.800, cut-off=915.38), demonstrating good discriminatory capacity. BMI and DAAT achieved moderate performance (AUC=0.795 and 0.775, respectively) with cut-offs of 28.65 kg/m² and 270.80, respectively **Table 5**.

In females, all indices demonstrated moderate diagnostic accuracy with generally lower AUC values. TGBMI remained the best performer (AUC=0.767, cut-off=842.95), followed by TGWC (AUC=0.751, cut-off=829.75). The reduced performance in females suggests sex-specific insulin resistance mechanisms that may require alternative diagnostic approaches or modified cut-off values **Table 5**.

Non-conventional indices (TG*BMI, WHtR, BRI, TG*WC, and LAP) consistently outperformed traditional measures across all three outcomes. Notably, BMI demonstrated varying cut-off values depending on the target condition: 27.24 kg/m² for CMR versus 29.28 kg/m² for insulin resistance, highlighting the importance of condition-specific thresholds. The superior performance of the combined biochemical-anthropometric indices (TG*BMI and TG*WC) underscores the value of integrating metabolic and adiposity markers for enhanced diagnostic accuracy **Table 4**.

## 4 Discussion

Cardiometabolic diseases are the leading cause of mortality worldwide, affecting ever-growing numbers of people across all ages and both sexes. We evaluated 15 conventional and non-conventional indices, -each noninvasive, cost-effective, and easy obtainable- based on anthropometric measurements, routine chemistry, or their combination in a national representative sample of Mexican adults. Waist-to-height ratio (WHtR), body-roundness index (BRI), deep abdominal adipose tissue index (DAAT), and triglyceride-based indices showed the strongest correlations with metabolic variables and the highest diagnostic power. ROC analysis confirmed that TG*BMI, WHtR, BRI, and TG*WC best detected obesity, whereas BRI, TG*WC, LAP, and DAAT most accurately identified the overall cardiometabolic risk. Although the optimal cut-off values differed by sex, discriminative performance remained consistently high in both men and women.

Data analysis revealed significant sex differences in both anthropometric and biochemical indices among 1,876 participants (754 men, 1.122 women). These differences reflect the underlying physiological, hormonal, and metabolic disparities between males and females that influence cardiometabolic risk assessment.

The most striking distinctions observed in the data reflect the well-documented sexual dimorphism in adipose tissue distribution patterns. Males demonstrated significantly higher waist circumference (98.49 vs. 95.61 cm, p<0.001) and DAAT (233.22 vs. 157.37, p<0.001), while females exhibited higher WHtR (0.62 vs. 0.59, p<0.001), BMI (29.97 vs. 28.65 kg/m², p<0.001), and BRI (6.14 vs. 5.38, p<0.001). These patterns are largely explained by sex-specific fat deposition regulated by sex hormones, particularly estrogen and testosterone. Research has established that males tend to accumulate more central/intra-abdominal (visceral) adipose tissue, whereas females typically store fat in subcutaneous and gluteal/femoral depots, reflecting android versus gynoid fat distribution patterns (85, 86). This distribution pattern is primarily mediated by estrogen’s complex effects on adipocyte metabolism, differentiation, and regional fat accumulation (87). Estrogen promotes subcutaneous fat accumulation while actively limiting visceral fat deposition through multiple coordinated molecular mechanisms. Estrogen increases the expression of antilipolytic α2A-adrenergic receptors exclusively in subcutaneous adipocytes, but not in visceral fat depots, thereby promoting fat storage in peripheral regions while facilitating lipolysis in central compartments (88). This receptor-mediated mechanism explains the preferential subcutaneous fat accumulation observed in premenopausal women and the subsequent redistribution toward visceral depots following menopause.

Additionally, estrogen enhances fatty acid oxidation through AMP-kinase (AMPK) phosphorylation in skeletal muscle tissue, which increases the sensitivity of acetyl-CoA carboxylase to palmitoyl-CoA inhibition, and subsequently promoting fatty acid oxidation. Furthermore, estrogen inhibits hepatic lipogenesis through direct transcriptional regulation of lipogenic enzymes, including fatty acid synthase (FASN) and stearoyl-CoA desaturase-1 (SCD-1), while simultaneously reducing cholesterol biosynthesis via HMG-CoA reductase suppression and increasing lipoprotein lipase activity, preferentially in the subcutaneous regions (85, 88).

The higher DAAT in males (233.22 vs. 157.37, p<0.001) directly reflects greater visceral adiposity. Current evidence indicates that visceral fat in men exhibits different metabolic activities than subcutaneous fat in women, including higher lipolytic rates and inflammatory cytokine production (85, 89). The hormonal environment significantly influences adipocyte progenitor cell differentiation (87). Interestingly, the lower BMI but higher DAAT in males illustrates how BMI alone fails to capture important sex differences in adipose tissue distribution relevant to cardiometabolic risk. This pattern suggests that males store proportionally more metabolically active visceral fat, whereas females accumulate more subcutaneous adipose tissue, which is typically less metabolically harmful (85, 90).

Biochemical indices revealed consistent patterns that aligned with the differences in adipose tissue distribution. Males demonstrate significantly higher triglycerides (204.72 vs. 161.60 mg/dL, p<0.001), triglyceride-glucose index (TG) (8.98 vs. 8.81, p<0.001), TG/HDL ratio (2.22 vs. 1.63, p<0.001), and atherogenic index of plasma (AIP) (0.23 vs. 0.13, p<0.001). These differences in lipid profiles stem from several interrelated factors. Visceral adiposity prevalent in males exhibits higher lipolytic activity, releasing free fatty acids directly into the portal circulation, which promotes hepatic triglyceride synthesis and VLDL production (89). Estrogen in females enhances HDL production and decreases hepatic lipase activity, contributing to higher HDL-C levels observed in women (46.80 vs. 43.69 mg/dL, p<0.001) (91).

Although hormonal factors play a crucial role in lipid metabolism, our study also examined the effectiveness of various anthropometric indices in assessing cardiometabolic risk. We found stronger correlations between WHtR and BRI (r=0.993), BMI and BRI (r=0.851), BMI and WHtR (r=0.846), and ABSI and WWI (r=0.816). These correlations reflect fundamental biochemical differences in the distribution and function of adipose tissues. Visceral adipose tissue, better captured by WHtR and BRI, is more metabolically active than subcutaneous fat, with higher rates of lipolysis releasing free fatty acids directly into portal circulation (92, 93). Among the biochemical indices, TG and AIP showed the strongest correlation, reflecting their shared biochemical foundations in lipid metabolism and insulin resistance pathways (94). The TG/HDL cholesterol ratio and AIP were also strongly correlated (r=0.841), which could be partly explained their mathematical relationship and by biochemical connections in lipoprotein particle size alterations occurring in insulin resistance (94).

We observed that biochemical measurements incorporating anthropometric parameters, such as TG*BMI and TG*WC, showed stronger correlations. Various anthropometric indices, such as BMI alone, serve as predictors of metabolic syndrome (MetS) irrespective of sex, while in females, the TG*WC indicator emerged as the most effective predictor of MetS. In individuals with MetS, anthropometric indicators can efficiently and rapidly identify those at risk of cardiovascular disease (95). BRI emerged as an indicator yielding optimal results, demonstrating strong correlations with various indicators and superior performance in the ROC curve for determining CMR and obesity. The ability of BRI to predict type 2 diabetes and cardiovascular risk stems from its accuracy in estimating visceral adiposity, which produces adipokines and inflammatory cytokines such as TNF-α, IL-6, and resistin, which impair insulin signaling pathways (96). In Jiangsu, eastern China, among adults, BRI functioned as a representative predictor of MetS and cardiovascular disease (95). The BRI was superior to BMI and waist circumference as predictors of MetS and its risk factors in the indigenous Peruvian population (97); superior to BMI in the detection, assessment, and progression of cardiovascular disease (CVD) and its risk factors; and closely associated with arterial stiffness in overweight and individuals with obesity (98, 99). BRI and WHtR exhibit similar abilities to predict MetS in adults, and both are effective in determining CVD (77, 98). The WHtR index has been reported as a reliable indicator of obesity and is also associated with cardiometabolic risk factors, such as hypertension, hypercholesterolemia, and elevated LDL (100). The WHtR was correlated with all anthropometric and biochemical indices, demonstrating stronger correlations with indices that utilize laboratory measurements and anthropometric variables, such as TG*WC and TG*BMI. The WHtR index has detected CMR in diverse populations, including individuals who are not with overweight or obesity, and is recommended for the early prevention of MetS. It represents a potentially valuable index due to its simplicity (92) and may predict cardiometabolic abnormalities, particularly in females (101).

Cardiometabolic risk aggregates modifiable factors—obesity, central adiposity, hypertension, hyperglycemia, dyslipidemia, smoking, inactivity, and poor diet—that jointly increase vascular events. Visceral fat is key; a higher waist-to-height ratio or visceral‐fat thickness associates with carotid intima-media thickening, an early atherosclerotic marker (102). Dyslipidemia and hyperglycemia boost mitochondrial ROS, activating NLRP3 inflammasomes and destabilizing plaques (103). Hypertension compounds damage by accelerating extracellular matrix remodeling and reducing NO bioavailability, leading to early arterial stiffening (102).

Physical inactivity is a major modifiable driver of CMR. A 2023 meta-analysis showed that aerobic and high-intensity interval exercise similarly slow carotid atherosclerosis by enhancing lipids and endothelial function (104). In contrast, diets rich in saturated fat and refined sugar aggravate dyslipidemia and insulin resistance and are associated with higher cardiovascular mortality (105).

An important limitation of ENSANUT is the absence of hip circumference, precluding waist-to-hip ratio (WHR) calculation. WHR is a robust marker of central adiposity that predicts cardiovascular events and mortality beyond BMI or waist circumference (106, 107). Larger hips attenuate, whereas smaller hips amplify, diabetes, and coronary-heart disease risks associated with a given waist size (108, 109). WHR is also independently related to subclinical myocardial injury (110). Without hip data, we cannot quantify the cardioprotective effect of gluteofemoral fat—typically greater in women—so CMR may be overestimated in participants with broad hips, and our sex-specific cut-offs may not extrapolate to groups with different fat patterns. Future Mexican surveys should therefore include hip measurements, enabling WHR and combined waist–hip models to be benchmarked against the non-conventional indices evaluated here and improving external validity.

These findings emphasize the necessity for integrated interventions targeting abdominal obesity, lipid/glucose homeostasis, and lifestyle modifications. Future public health strategies should prioritize visceral adiposity reduction through community-based exercise programs and dietary education, leveraging noninvasive indices such as WHtR and BRI for early risk stratification in resource-limited settings.

Exercise and organized sports are the most accessible, cost-effective first-line interventions for lowering cardiometabolic risk. Alongside the screening indices (WHtR, BRI, and DAAT), regular physical activity remains central to risk management. Recent evidence shows that sports participation markedly slows cardiometabolic risk trajectories, with pronounced benefits in women. Marques-Elias et al. (2021) reported that lifelong sports engagement plus current activity reduced obesity and improved metabolic profiles in female workers (111).

The present analysis indicates that simple, inexpensive indices—particularly WHtR, BRI, DAAT, TGBMI, and TGWC—outperform traditional metrics for detecting adiposity, insulin resistance, and global cardiometabolic risk in Mexican adults. Integrating these tools into routine primary care and community-based programs, and validating them prospectively alongside hip-circumference measures, could substantially advance the early detection and prevention of cardiometabolic disease in resource-constrained settings.

## 5 Conclusions

This nationally representative ENSANUT analysis demonstrates that several non-conventional indices—most notably TGBMI, TGWC, LAP, BRI, DAAT, and WWI—surpass traditional metrics (BMI, WC, WHtR) in identifying obesity, insulin resistance, and overall cardiometabolic risk in Mexican adults. As these indices rely on inexpensive anthropometric and basic biochemical measurements, they offer practical screening tools for resource-limited settings.

### 5.1 Limitations of the study

The main strength of this study is its use of rigorously collected, nationally representative ENSANUT data. Nevertheless, this study has several limitations must be considered. (i) Its cross-sectional design precludes causal inference; therefore, temporal relationships between the indices and incident cardiometabolic events cannot be established. (ii) Listwise deletion of incomplete records reduces the analytical sample size and may introduce selection bias, thereby limiting generalizability. (iii) The Body Roundness Index yielded an AUC of 1.000 for cardiometabolic risk, an implausibly perfect value that probably reflects sample-specific overfitting rather than true diagnostic perfection; replication in independent cohorts is needed. (iv) The database lacked hip circumference data, preventing the calculation of the waist-to-hip ratio (WHR), a widely validated marker of central adiposity and CMR. (v) Residual confounding by unmeasured lifestyle factors, such as diet quality or physical activity, cannot be excluded.

### 5.2 Future directions

Prospective cohort studies should track incident diabetes, hypertension, and cardiovascular events to confirm the predictive utility of these non-conventional indices and calibrate time-dependent cut-offs. Randomized interventions that reduce visceral adiposity—e.g., high-intensity interval training or energy-restricted diets—could test whether index improvements translate into measurable reductions in CMR. Future surveys should include hip circumference measurements to permit WHR calculations and enable direct comparisons with conventional markers. Finally, external validation across different Mexican regions, other Latin-American populations, and varied age strata is essential to refine sex-specific thresholds, assess reproducibility, and facilitate the integration of these indices into public health screening programs.

## Data Availability

Publicly available datasets were analyzed in this study. This data can be found here: https://ensanut.insp.mx/encuestas/ensanutcontinua2022/descargas.php.
